# Oleic Acid Produced by a Marine *Vibrio* spp. Acts as an Anti-*Vibrio parahaemolyticus* Agent

**DOI:** 10.3390/md9102155

**Published:** 2011-10-24

**Authors:** Yanett Leyton, Jorge Borquez, José Darias, Mercedes Cueto, Ana R. Díaz-Marrero, Carlos Riquelme

**Affiliations:** 1 Microbial Ecology Laboratory, Faculty of Marine Resources, Antofagasta University, Antofagasta 1310000, Chile; E-Mail: criquelme@uantof.cl; 2 Doctorate Applied Science, Mention Coastal Marine Systems, Faculty of Marine Resources, Antofagasta University, Antofagasta 1310000, Chile; 3 Laboratory of Natural Products, Department of Chemistry, Antofagasta University, Antofagasta 1310000, Chile; E-Mail: jborquez@uantof.cl; 4 Institute of Natural Products and Agrobiology, CSIC, La Laguna, Tenerife 38206, Spain; E-Mails: jdarias@ipna.csic.es (J.D.); mcueto@ipna.csic.es (M.C.); adiaz@ipna.csic.es (A.R.D.-M.)

**Keywords:** bioactive marine products, inhibition, *Vibrio parahaemolyticus*, oleic acid

## Abstract

It is known that some strains of *Vibrio parahaemolyticus* are responsible for gastroenteric diseases caused by the ingestion of marine organisms contaminated with these bacterial strains. Organic products that show inhibitory activity on the growth of the pathogenic *V. parahaemolyticus* were extracted from a *Vibrio* native in the north of Chile. The inhibitory organic products were isolated by reverse phase chromatography and permeation by Sephadex LH20, and were characterized by spectroscopic and spectrometric techniques. The results showed that the prevailing active product is oleic acid, which was compared with standards by gas chromatography and high-performance liquid chromatography (HPLC). These active products might be useful for controlling the proliferation of pathogenic clones of *V. parahaemolyticus*.

## 1. Introduction

The marine ecosystem covers around 70% of the surface of the planet, and because of its biodiversity of macro- and microorganisms it can be thought of as an important source for the extraction of bioactive marine natural products (BMNP). These BMNPs have been studied for use in the pharmaceutical industry, to obtain antitumoral drugs, antivirals, antifungals, antihelmintics, antiinflamatories, analgesics, immunoregulators, and food supplements, among others [1,2].

Since the 1960s there has been increasing research on marine natural products [3]. Studies of the chemistry and biological activity of organisms like sponges, coelenterates, and echinoderms have shown that they contain a large amount and variety of secondary metabolites with chemical structures different from those found in land organisms [4]. Since 1990, the bioactive metabolites discovered in marine bacteria have increased exponentially [5]. Most of the bioactive agents have been isolated from *Streptomyces*, *Alteromonas*/*Pseudoalteromonas*, *Bacillus*, *Vibrio*, *Pseudomonas*, and *Cytophaga* obtained from seawater, sediments, marine algae, and invertebrates that produce quinones, polyenes, macrolides, alkaloids, peptides, and to a smaller extent terpenoids [3]. It has been determined that some products obtained from marine bacteria have bioactive effects against other marine bacteria [6,7].

In the 1940s research was centered on secondary metabolites (SM), organic compounds that are produced when cellular growth stops and are synthesized as mixtures of chemically related compounds, with a huge variety of chemical structures, as a consequence of the diversification and branching of their biosynthetic routes [8]. The factors that trigger the production of SMs are not well known, but they can be produced when some nutrient in the environment is in limited supply, such as nitrogen, carbon, or phosphorus, altering the production of primary metabolites, giving rise to enzyme inductors that lead to SMs. In the aquaculture industry, the use of antibiotics has been forbidden because they have toxic effects on human health, they cause a negative impact on the environment, and their frequent use generates metabolic changes in bacteria that creates resistance to them. In aquaculture this resistance to antibiotics generates interest in searching for alternative antibiotics, as well as to control “vibriosis”, a common disease caused by bacteria in marine cultures around the world, affecting the cultivation of mollusks [9], crustaceans [10], and fish [11]. Chile has reported outbreaks of food intoxication caused by the presence of *Vibrio parahaemolyticus* due to the ingestion of raw or undercooked mollusks, fish and shrimp. In the last decade, there have been three such major outbreaks in Chile (Antofagasta 1998 with 300 cases, Puerto Montt 2004–2007 with more than 7000 cases) [12]. Isnansetyo *et al.* [11] propose the use of strain S2V2 as a biological control alternative, a bacterium that showed inhibiting activity against 68% of a total of 28 pathogenic vibrios analyzed, but the challenge remains to purify and elucidate the chemical structure of its inhibiting metabolites. Fatty acids are nontoxic compounds that show bactericidal effects [13,14] and have been investigated for many years [15]. These fatty acids have been incorporated in foods with the purpose of preventing the action of human pathogens like *Salmonella*, *Listeria* and *Staphylococcus* [14]. Singh *et al.* [16] isolated a fatty acid from the cyanobacteria *Lyngbya majuscula* with activity against *Candida albicans*. Parés and Juárez [8] propose that the antibiotic activity of the SMs generated by the bacteria is based on their ability to inhibit essential primary metabolic processes.

The search for new BMNPs with the ability to inhibit the growth of pathogenic bacteria can promote the development of new sources of antibiotics compatible with the environment for use in marine cultures [17] and in this way solve the problems of bacterial contamination that have large economic effects on the extractive industry of marine resources. In this paper we report the extraction and purification of an anti-*V. parahaemolyticus* molecule isolated from a marine *Vibrio*.

## 2. Results

**Figure 1:**
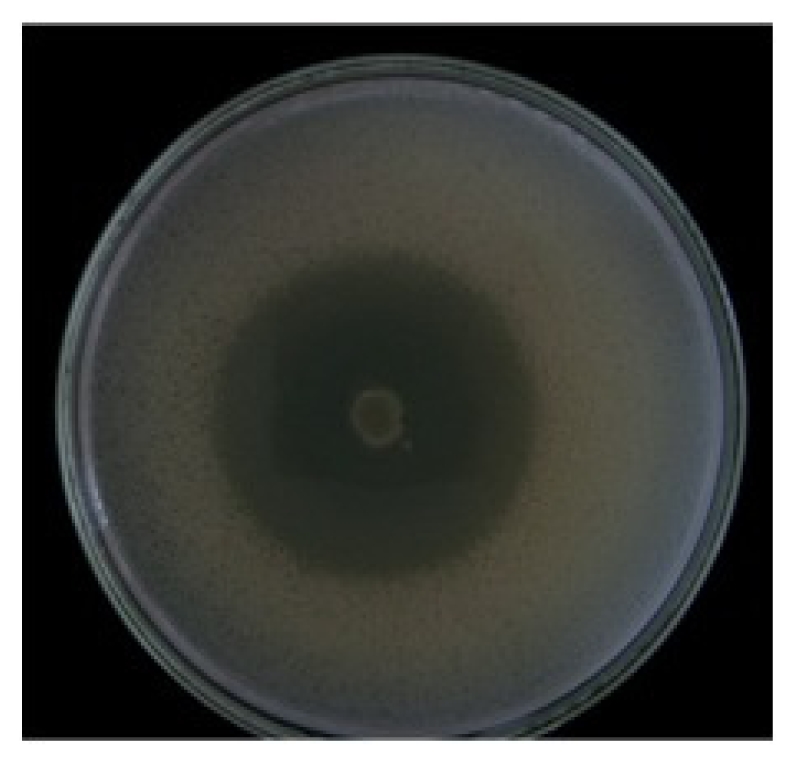
Identification of the inhibitory activity of the environmental strain against the human pathogen *V. parahaemolyticus* by the Dopazo method. The *V. parahaemolyticus* strains was inoculated above the environmental strain together with a second layer of semisolid agar.

The environmental strain used in this work was isolated from scallop’s culture system and was selected by a strong and stable inhibitory activity against pathogenic bacteria. In this work we reported for the first time, the ability of this strain to inhibit the growth of the pathogen *V. parahaemolyticus*, this was evidenced by the method of double layer agar (Dopazo) showing an inhibition halo of 35 mm (Figure 1). These inhibition values were reproducible and no significant differences between the replicates were observed (*P* > 0.05). The results of the molecular characterization using the sequencing of gene 16s rRNA indicated that the environmental strain corresponded to the genus *Vibrio* (according to the GenBank database), and the closest relative in GenBank resulted *Vibrio* sp. 52B8 (accession number JF346764) with 100% similarity.

**Figure 2:**
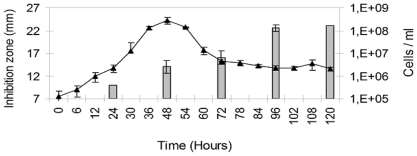
Evaluation of the growth and production of antibacterial substance of the environmental strain. Bars indicate the size of the inhibition zone (in mm) and the curve indicates the bacterial counts in cell/mL.

Data obtained from the experimental growth of the environmental strain and extraction of their bioactive products showed that the organic production increased significantly between 96 and 120 h compared to 24, 48 and 72 h. The best inhibition effect was recorded when bacteria entered the stationary phase (Figure 2).

Evaporation of the solvent, after obtaining different fractions of the extract, resulted in a purified residue, whose chromatographic and spectroscopic data (IR, MS and 13C and 1H NMR) when compared to the database were identified as a monounsaturated fatty acid of long chain called Oleic Acid (OA). The total yield of the active organic product (without fractionation) obtained from the environmental strain was about 264 mg/80 L of culture.

## 3. Discussion

The results of this work suggest that the environmental strain has similitude (100%) with the strain *Vibrio* sp. 52B8 (according GenBank database). Jorquera *et al.* [18] also reported activity inhibitory of this bacterium against *V. anguillarum*-VAR, *V. parahaemolyticus*, and *V. splendidus*, identifying an aliphatic hydroxyl ether as the inhibiting molecule produced by these pathogenic vibrios. The importance of our results is that we obtained a better purification of the inhibitory product and we have identified oleic acid as the active product. Also, Jorquera *et al.* [18] used the same organic solvent for its extraction, with the difference that the bioactive substances were obtained only from the supernatant of the culture, while in our work we obtained the bioactive substances from the bacteria and the culture supernatant.

The antibacterial action of fatty acids is commonly attributed to long chain unsaturated fatty acids like oleic, linoleic, and linolenic, and their mechanism of action is to inhibit fatty acid synthesis [13]. Fatty acids are known not to inhibit Gram negative bacteria like *Escherichia coli* [19], and this great difference can be a consequence of the impermeability of the outer membrane of Gram negative bacteria, which acts as a barrier against hydrophobic substances [10]. In our work, we isolated OA from a bacterial strain of the genus *Vibrio*, which has the particularity of inhibiting the growth of *V. parahaemolyticus*. The prevalence of this *Vibrio* in the marine ecosystem of the Bay of Antofagasta should be evaluated because it may account for the low occurrence of the pathogenic clone *V. parahaemolyticus*, with only isolated cases since an epidemic outbreak in 1998 [20], so these metabolites may be helping, together with other abiotic factors, to control this pathogen. Even though the relation between the structure and the antimicrobial activity of OA is not clear, it seems that the number and position of the double bonds, together with having a hydrophilic head and a hydrophobic tail may influence the antimicrobial activity, affecting the bipolar membrane of the bacterial cell wall. OA is known to have bactericidal activity against important pathogenic microorganisms [21,22], including *Staphylococcus aureus* [23], *Helicobacter pylori* [22], and *Mycobacteria* [21], and it has been suggested that it provides numerous benefits to human health because its moderate use can lower cholesterol levels and reduce atherosclerosis [24]. It further participates in the synthesis of membrane phospholipids and contributes to cell membrane physiology in mechanisms, such as signal transduction and cellular proliferation [25]. Lunde *et al.* [26] suggest that the antibacterial effect of oleic acids can be related to its ability to penetrate through the cell membranes of bacteria and fungi, causing their death by altering the normal function of the cell membrane. Huang *et al.* [27] reported for the first time, extensive antibacterial activity of oleic acids against oral microorganisms, including *Streptococcus mutans*, *Aggregatibacter actinomycetemcomitans*, *Candida albicans*, *Porphyromonas gingivalis*, *Fusobacterium nucleatum*, and *Streptococcus gordonii*, suggesting that more studies are needed to show that oleic acids can be used as complementary biomolecules to be incorporated in humans through various vectors to attack oral infections, caries, and/or periodontal disease *in situ*, using different methods like chewing gum, toothpaste, juices, and milk. Cardoso *et al.* [28] propose that oleic acid modulates wound inflammation and increases the *in vivo* repair response in skin lesions, and they suggest that it can be used as treatment for skin injuries, especially for burns, diabetes, or ulcers.

## 4. Experimental Section

### 4.1. Origin of Bacterial Strains

The study dealt with the isolated pathogenic bacteria *Vibrio parahaemolyticus* strain PM48.5 [17] and the environmental strain [29] obtained from the strain collection of the Laboratorio de Ecología Microbiana of the Universidad de Antofagasta. The bacteria were kept in a strain collection in Tryptone Soy Agar culture medium (TSA Oxoid Ltd., Basingstoke, Hampshire, England) under axenic conditions at 20 ± 1 °C and frozen in cryobeads.

### 4.2. Molecular Characterization of the Environmental Strain

The genomic DNA of the environmental strain was extracted using the method described by [30]. Then gene 16s rRNA was amplified by PCR, using universal primers, performing a first amplification with primers 27F and 1542R previously described by [31]. Three processes were then performed for sequencing completely the 16s rRNA using the primer 358F, 907 R and the 1492R. The PCR product was purified with the purification kit (UltraClean™15 DNA, MoBio Laboratories, CA, USA) according to the manufacturer’s instructions, and its DNA was sequenced (Macrogen Inc., Korea). The alignments were made with Clustal W [32] on the Bioedit program and the sequence was compared with those that were available in the GenBank database.

### 4.3. Inhibition Tests

The inhibition tests were carried out by the “double layer” method [33], inoculating 10 μL (7.1 × 10^5^ cells/mL) of the environmental strain from an overnight culture in the center of a Petri dish with Müller Hinton medium (Difco), incubating at 20 °C for 48 h. After that time the macro colony formed was exposed to chloroform vapors for 45 min, then a second layer of semisolid agar previously inoculated with the pathogenic bacteria *V. parahaemolyticus* (2.3 × 10^4^ cells/mL) was added and it was incubated at 20 °C for 48 h. The presence of a well defined inhibition halo around the macro colony was considered as antibacterial activity. The study was made in triplicate and the degree of inhibition was determined measuring the diameter of the halo, considering values greater than 5 mm as strong inhibition according to [34].

### 4.4. Growth of the Environmental Strain and Extraction of Its Bioactive Products

The environmental strain was inoculated in minimum medium M9 [35] at the initial concentration of 1 × 10^7^ cells/mL at 20 °C. Bacteria abundances (cells mL^−1^) were counted after of 6, 12, 24, 30, 36, 48, 54, 60, 72, 78, 90, 96, 102, 108, and 120 h, staining the bacteria with fluorochrome 4′,6-diamino-2-phenylindol (DAPI) [36] and observing under an epifluorescence microscope at 100× (Olympus BN-2) equipped with a DM400 dichroic mirror and UG1 excitation and L420 absorption filters. In parallel, the organic products were extracted after 24, 48, 72, 96, and 120 h of cultivation, adding 150 mL of the ethyl acetate per liter of culture (bacteria + supernatant). The organic phase was recovered and it was left for 20 min over anhydrous sodium sulfate (Merck, Germany), filtered through filter paper, concentrated to dryness in a rotavapor at 45 °C, and lyophilized for 24 h to remove the moisture. The initial sample or extract was kept at −20 °C until its use. Every hour of sampling bioassays were carried out by the diffusion filter method adding 2 mg/product filter. The pathogenic *V. parahaemolyticus* was previously laid in extended a concentration of (1 × 10^5^ cells/mL). The plates were incubated for 48 h at 20 °C.

### 4.5. Purification and Characterization of the Oleic Acid

The environmental strain was cultivated in 80 L of M9 minimum liquid medium for 96 h at an initial concentration of 1 × 10^7^ cells/mL at 20 °C, and the same extraction method mentioned under point 4.4 was used. The ethyl acetate extract (350 mg) was separated depending on their polarity by means of reverse phase chromatography (RPC) in a chromatographic column compacted with silica gel (100 C18-Reversed phase) to a height of 10 cm. The extract was added to the column adsorbed on the same silica gel used in the column. The mobile phase was distilled water (100 mL) as an initial fraction, then mixtures (100 mL) of distilled water and methanol in 3:1, 3:2, 2:3 and 1:4 ratios, and finally 100 mL of the solvents methanol, dichloromethane, and methanol. From this procedure were obtained 8 fractions: 1 (16.9 mg), 2 (1.6 mg), 3 (14.4 mg), 4 (37.5 mg), 5 (21.9 mg), 6 (120.6 mg), 7 (37.7 mg) and 8 (12.8 mg) them dried in a rotavapor at 45 °C and lyophilized for 24 h. From these fractions the number 6 showed inhibitory activity against *V. parahaemolyticus* and them this fraction was eluted in methanol 100% and added to a column using Sephadex LH-20 (Pharmacia Fine Chemicals ref. 17-090-01), and a hexane/dichloromethane/methanol mixture in a 3:1:1 mobile phase. From this procedure were obtained 8 fractions: 1 (9.7 mg), 2 (6.2 mg), 3 (76.9 mg), 4 (11.6 mg), 5 (5 mg), 6 (2.2 mg), 7 (3.8 mg) and 8 (5.4 mg) were dried in a rotavapor at 45 °C and lyophilized for 24 h. The active fraction 3 (76.9 mg) pure enough (98%) was unambiguously identified as oleic acid, by comparing their mass spectra and ^1^H and ^13^C NMR to those of authentic compound.

### 4.6. Statistical Analysis

The experiments were designed to evaluate the effects of the antibacterial products from the environmental strain on the growth of *V. parahaemolyticus*. Before ANOVA, the data were evaluated in terms of their fulfillment of the assumptions of variance homogeneity (Bartlett’s test), normal data distribution (Kolmogorov-Smirnov test), normality of the residuals (Anderson-Darling test), independence and linearity of the model using the MINITAB 14 statistical software. The data were then analyzed with one-way ANOVA [37].

## 5. Conclusions

In this study, the isolation of an environmental strain belonging to the genus *Vibrio*, with antibacterial effect of *V. parahaemolyticus* growth, was reported. This could have profound ecological implications in the aquatic ecosystems. The oleic acid produced by this *Vibrio* strain could be useful as a biocontrol against *V. parahaemolyticus* and might represent a contribution not only in the aquaculture industry, but also at the clinical level. However, optimizing the production of the active organic product of this bacterial strain is a challenge that must be considered in order to use this compound to contrast the pathogenic action of *V. parahaemolyticus*.

## References

[1] Duque B (1998). Busqueda de compuestos bioactivos a partir de organismos marinos del caribe colombiano. Rev. Acad. Colomb. Cienc.

[2] Newman DJ, Cragg GM (2004). Marine natural products and related compounds in clinical and advanced preclinical trials. J. Nat. Prod.

[3] AL-Zereini W (2006). Natural Products from Marine Bacteria. Ph.D. Thesis.

[4] Márquez D, Galeano E, Martínez A (2004). Productos naturales con actividad antimicrobiana. Parte II. Vitae Rev. Fac. Quím. Farm.

[5] Blunt JW, Copp BR, Munro MHG, Northcote PT, Prinsep MR (2006). Marine natural products. Nat. Prod. Rep.

[6] Zapata M, Silva S, Luza Y, Wilkens M, Riquelme C (2007). The inhibitory effect of biofilms produced by wild bacterial isolates to the larval settlement of the fouling ascidia *Ciona intestinalis* and *Pyura praeputialis*. Electron. J. Biotechnol.

[7] Zhang Y, Mu J, Gu X, Zhao C, Wang X, Xie Z (2009). A Marine sulfate-reducing bacterium producing multiple antibiotics: Biological and chemical investigation. Mar. Drugs.

[8] Parés R, Juárez A (1997). Bioquímica de los Microorganismos.

[9] Cai J, Li J, Thompson KD, Li C, Han H (2007). Isolation and characterization of pathogenic *Vibrio parahaemolyticus* from diseased post-larvae of abalone *Haliotis diversicolor supertexta*. J. Basic Microbiol.

[10] Balcazar JL, Rojas-Luna T, Cunningham D (2007). Effect of the addition of four potential probiotic strains on the survival of pacific white shrimp (*Litopenaeus vannamei*) following immersion challenge with *Vibrio parahaemolyticus*. J. Invert. Pathol.

[11] Isnansetyo A, Istiqomah I, Muhtadi, Sinansari S, Hernawan RK, Triyanto, Widada J (2009). A potential bacterial biocontrol agent, strain S2V2 against pathogenic marine *Vibrio* in aquaculture. World J. Microbiol. Biotechnol.

[12] Harth E, Matsuda L, Hernández C, Rioseco ML, Romero J, González-Escalona N, Martínez-Urtaza J, Espejo R (2009). Epidemiology of *Vibrio parahaemolyticus* Outbreaks, Southern Chile. J. Infect. Dis.

[13] Zheng CJ, Yoo JS, Lee TG, Cho HY, Kim YH, Kim WG (2005). Fatty acid synthesis is a target for antibacterial activity of unsaturated fatty acids. FEBS Lett.

[14] Lekogo BM, Coroller L, Mathot AG, Mafart P, Leguerinel I (2010). Modelling the influence of palmitic, palmitoleic, stearic and oleic acids on apparent heat resistance of spores of *Bacillus cereus* NTCC 11145 and *Clostridium sporogenes* Pasteur 79.3. Int. J. Food Microbiol.

[15] Nieman C (1954). Influence of trace amounts of fatty acids on the growth of microorganisms. Bacteriol. Rev.

[16] Singh IP, Milligan KE, Gerwick WH (1999). Tanikolide, a toxic and antifungal lactone from the marine cyanobacterium *Lyngbya majuscula*. J. Nat. Prod.

[17] Leyton Y, Riquelme C (2010). Marine *Bacillus* spp. Associated with the egg capsule of *Concholepas concholepas* (common name “loco”) have an inhibitory activity toward the pathogen *Vibrio parahaemolyticus*. Microb. Ecol.

[18] Jorquera M, Riquelme C, Loyola L, Muñoz L (1999). Production of bactericidal substance by a marine *Vibrio* isolated from cultures of the scallop *Argopecten purpuratus*. Aquac. Int.

[19] Sun CQ, O’Connor CJ, Roberton AM (2003). Antibacterial actions of fatty acids and monoglycerides against *Helicobacter pylori*. FEMS Immunol. Med. Microbiol.

[20] Fuenzalida L, Armijo L, Zabala B, Hernández C, Rioseco ML, Riquelme C, Espejo R (2007). *Vibrio parahaemolyticus* strains isolated during investigation of the summer 2006 seafood related diarrhea outbreaks in two regions of Chile. Int. J. Food Microbiol.

[21] Seidel V, Taylor PW (2004). *In vitro* activity of extracts and constituents of Pelagonium against rapidly growing mycobacteria. Int. J. Antimicrob. Agents.

[22] Sun CQ, O’Connor CJ, Roberton AM (2003). Antibacterial actions of fatty acids and monoglycerides against *Helicobacter pylori*. FEMS Immunol. Med. Microbiol.

[23] Farrington M, Brenwald N, Haines D, Walpole E (1992). Resistance to desiccation and skin fatty acids in outbreak Straits of methicillin-resistant *Staphylococcus aureus*. J. Med. Microbiol.

[24] Nicolosi RJ, Woolfrey B, Wilson TA, Scollin P, Handelman G, Fisher R (2004). Decreased aortic early atherosclerosis and associated risk factors in hypercholesterolemic hamsters fed a high or mid oleic acid oil compared to a high-linoleic acid oil. J. Nutr. Biochem.

[25] Ziboh VA, Miller CC, Cho Y (2000). Metabolism of polyunsaturated fatty acids by skin epidermal enzymes: Generation of anti-inflammatory and antiproliferative metabolites. Am. J. Clin. Nutr.

[26] Lunde CS, Hartouni SR, Janc JW, Mammen M, Humphrey PP, Benton BM (2009). Telavancin disrupts the functional integrity of the bacterial membrane through targeted interaction with the cell wall precursor lipid II. Antimicrob. Agents Chemother.

[27] Huang C, George B, Ebersole JL (2010). Antimicrobial activity of *n*-6, *n*-7 and *n*-9 fatty acids and their esters for oral microorganisms. Arch. Oral Biol.

[28] Cardoso CR, Favoreto S, Oliveira LL, Vancima JO, Barbana GB, Ferraza DB, Silva JS (2010). Oleic acid modulation of the immune response in wound healing: A new approach for skin repair. Immunobiology.

[29] Riquelme C, Araya R, Vergara N, Rojas R, Guaita M, Candia M (1997). Potential of probiotic strains in the culture of the Chilean scallop *Argopecten purpuratus* (Lamarck, 1819). Aquaculture.

[30] Sambrook J, Fritsch EF, Maniatis T (1989). Molecular Cloning: A Laboratory Manual.

[31] Brosius J, Dull TJ, Sleeter DD, Noller HF (1981). Gene organization and primary structure of a ribosomal RNA operon from *Escherichia coli*. J. Mol. Biol.

[32] Thompson D, Higgins G, Gibson J (1994). CLUSTAL W: Improving the sensitivity of progressive multiple sequence alignment through sequence weighting, position-specific gap penalties and weight matrix choice. Nucleic Acids Res.

[33] Dopazo CP, Lemos ML, Bolinches J, Barja JL, Toranzo AE (1988). Inhibitory activity of antibiotic-producing marine bacteria against fish pathogens. J. Appl. Bacteriol.

[34] Avendaño-Herrera R, Lody M, Riquelme CE (2005). Production of inhibitory substances among bacterial biofilms on marine substrates. Rev. Biol. Mar. Oceanogr.

[35] Gerhardt P, Murray RGE, Wood WA, Krieg NR (1994). Methods for General and Molecular Bacteriology.

[36] Porter K, Feig Y (1980). The use of DAPI for identifying and counting aquatic microflora. Limnol. Oceanogr.

[37] Zar J (1994). Biostatistical Analysis.

